# Supplementary material for: Surface Microbiome Profiling of Dental Elevators Using Third‐Generation Sequencing: Implications for Infection Control in Dental Practice
Source: Microbiologyopen. 2025 Dec 8;14(6):e70178. doi: 10.1002/mbo3.70178 (PMC12685768; doi:10.1002/mbo3.70178)
Supplement: Supplementary file 1 — customer_backup. [file MBO3-14-e70178-s001.zip › customer_backup/customer_backup/taxa_summary/krona/groups/treat/M02.Krona.html]

Javascript must be enabled to view this page.

members
magnitude
magnitudeUnassigned

M02.krona

49624

49623

65

0

0

0

0

0

0

0

0

0

0

0

0

0

0

0

65

65

65

65

19

1

6

0

4

22

3

3

7

0

0

0

0

0

0

0

0

0

0

0

0

0

0

160

70

0

0

0

0

0

0

0

0

0

0

0

0

0

0

0

0

0

70

0

0

0

1

1

1

0

0

38

3

3

35

35

3

3

3

6

0

0

1

1

5

5

0

0

0

22

17

17

5

5

90

0

0

0

0

0

0

0

90

15

15

15

16

16

16

4

4

4

10

10

10

45

45

45

196

165

165

1

1

1

164

115

113

2

34

3

30

0

1

15

15

0

0

0

0

0

0

0

0

0

0

0

0

0

0

0

0

0

0

0

0

0

0

0

0

0

0

0

0

0

0

0

0

0

0

0

0

0

0

0

0

0

0

0

0

0

0

0

0

0

0

24

24

1

0

0

0

0

0

0

0

0

0

0

0

0

1

1

0

0

0

0

0

0

0

0

0

23

0

0

0

0

0

23

23

7

0

0

0

0

0

0

0

0

0

0

0

0

0

0

0

0

0

0

0

7

7

7

7

0

0

0

0

0

0

0

0

0

0

0

0

0

0

0

1

1

1

1

1

1

0

0

0

0

0

0

0

0

0

0

0

0

0

0

0

0

0

0

0

0

0

0

0

0

0

0

225

45

45

45

2

2

20

20

6

0

6

17

8

9

1

1

1

1

1

179

179

179

11

10

1

156

0

0

54

0

102

6

6

6

4

2

460

96

96

0

0

0

96

96

96

2

2

2

2

2

0

0

0

0

0

0

0

0

0

0

0

362

219

208

184

39

132

13

24

4

16

4

0

11

11

10

1

12

10

10

10

2

2

2

0

0

124

3

0

0

0

0

3

0

3

0

0

0

0

0

0

0

0

0

0

0

0

0

0

0

0

121

121

21

100

7

7

0

0

0

0

1

1

6

1

5

0

0

0

0

0

0

0

9680

4375

0

0

0

0

76

1

1

1

74

2

2

0

4

4

0

0

0

68

3

65

0

1

1

1

150

150

83

1

82

58

58

9

9

41

0

0

0

41

1

1

1

1

2

2

37

37

0

0

0

60

60

1

0

1

0

59

0

0

6

53

0

0

0

0

0

0

0

451

451

0

0

30

1

1

3

18

7

0

0

0

0

19

19

3

3

0

0

0

0

0

0

50

0

1

2

30

4

1

12

0

0

99

4

58

26

11

3

3

0

212

4

0

17

0

1

2

49

0

0

11

4

1

8

20

0

0

2

5

43

1

21

0

0

9

8

0

2

4

0

0

35

10

6

0

19

0

0

0

0

0

0

0

0

0

1808

61

0

0

28

0

28

0

0

0

0

0

0

0

0

33

8

5

20

0

0

0

52

3

2

1

41

27

4

0

5

1

0

2

2

8

8

3

3

3

0

0

0

2

2

2

5

5

5

0

0

0

297

0

0

0

0

1

0

1

114

106

8

0

0

0

3

3

0

0

0

0

0

3

3

98

1

76

5

13

1

0

2

0

0

78

4

0

0

1

13

10

3

3

0

32

3

9

0

0

96

35

35

3

3

33

33

21

21

4

4

0

0

0

29

29

29

0

0

0

5

5

5

79

16

2

3

11

0

0

0

0

0

63

8

49

0

6

66

57

57

0

9

9

0

447

12

5

7

45

45

138

138

6

5

1

13

13

1

1

6

6

2

2

224

14

210

56

56

2

54

610

179

179

1

1

1

1

13

0

0

13

0

0

315

14

44

6

1

90

1

14

0

0

6

131

8

0

0

0

0

33

9

0

14

10

0

0

0

0

12

12

56

56

4

4

0

0

4

4

61

8

6

6

2

2

53

15

1

6

3

5

7

4

3

6

1

0

1

4

11

11

14

14

0

0

3

3

3

3

0

0

0

0

0

0

0

0

0

0

0

0

0

0

0

0

5

5

5

5

0

0

0

0

1

1

1

1

0

0

0

0

0

0

0

0

0

0

0

0

0

0

0

0

0

0

0

1365

1365

0

0

3

1

2

1259

1259

9

0

0

2

2

4

0

1

0

2

2

40

19

19

2

1

1

46

8

22

16

0

0

5

5

0

0

0

0

12

3

3

3

0

0

0

2

2

2

2

2

2

0

0

0

1

1

1

0

0

0

1

1

1

3

3

3

338

338

0

0

0

0

0

0

0

0

0

0

0

0

0

0

0

0

0

0

0

0

0

0

0

0

0

14

13

1

0

0

1

1

29

2

0

27

0

0

271

35

69

167

0

0

1

1

22

1

21

0

5305

0

0

0

0

0

0

0

0

0

0

0

0

11

11

11

11

0

0

0

3308

0

0

0

0

0

0

0

0

0

0

0

0

0

0

0

0

0

0

0

0

0

0

0

0

14

14

14

27

27

27

52

52

52

0

0

1773

4

4

1

1

1581

1581

84

22

10

33

19

103

0

0

0

0

0

33

1

34

2

9

7

0

6

0

0

11

39

1

1

13

13

4

1

1

2

0

0

2

2

19

0

19

2

2

2

58

0

0

1

1

0

0

18

0

0

18

38

38

0

0

1

1

144

35

35

0

0

31

11

20

78

0

78

0

64

64

64

1056

0

0

3

0

3

0

7

7

18

18

0

0

41

7

17

17

34

20

14

0

0

31

2

29

37

37

31

1

30

5

4

0

1

5

5

3

0

3

840

840

1

1

79

22

22

57

0

57

91

69

35

27

8

32

32

2

2

22

22

0

22

0

0

0

0

53

53

53

53

0

0

0

0

0

0

0

48

48

48

0

48

1

1

1

1

0

0

0

0

0

0

0

0

0

0

0

0

0

0

0

64

1

1

1

20

1

1

0

0

0

0

0

0

0

19

0

9

0

10

0

0

0

0

0

0

0

0

0

0

0

0

0

0

0

13

13

13

0

0

0

7

7

0

0

6

1

23

0

0

0

0

0

0

0

22

0

22

0

1

1

0

0

0

0

0

0

0

0

0

0

0

0

0

24

24

0

0

0

0

24

24

0

0

0

0

0

0

0

0

0

0

0

0

0

0

0

0

0

0

0

0

0

0

0

1590

6

5

5

1

1

0

0

0

3

0

0

0

3

3

0

0

0

994

959

959

5

5

22

22

8

8

21

21

21

0

373

373

373

0

0

0

32

32

32

161

0

0

0

0

0

0

0

0

32

20

12

0

0

129

109

20

0

0

0

0

0

0

0

0

0

0

0

16

16

16

11

0

2

0

0

0

3

0

0

0

0

0

0

0

0

0

0

0

0

43

14

1

1

3

3

0

4

4

5

5

1

0

1

29

0

0

1

1

0

0

2

1

1

20

10

0

10

0

6

0

0

0

0

0

0

0

0

0

3

1

2

0

0

0

0

0

0

0

0

0

0

0

0

0

0

0

0

0

0

0

0

0

0

0

56

56

56

1

43

12

0

0

0

0

0

0

0

0

0

0

0

0

0

0

0

0

0

0

0

0

0

0

0

0

0

0

0

0

0

0

0

0

0

0

0

0

0

0

0

0

0

0

0

0

256

154

154

0

0

0

154

21

21

8

8

1

1

5

5

19

8

11

10

10

0

0

90

89

0

1

102

0

0

0

0

0

0

0

1

0

0

0

0

0

0

1

1

1

41

20

5

5

0

0

0

0

2

1

1

13

3

0

10

1

1

1

0

0

0

0

10

0

0

0

10

0

0

9

1

10

4

4

6

6

0

0

0

0

60

60

60

2

1

29

28

0

0

0

0

0

0

0

0

0

0

0

0

0

0

0

0

0

0

0

0

0

27

27

27

27

27

5

9

1

12

0

0

0

0

0

0

4104

0

0

0

0

0

4101

276

4

4

4

269

2

1

1

65

11

15

39

6

2

0

2

2

54

0

54

0

6

6

0

0

79

11

10

4

10

8

22

3

10

1

18

3

1

8

1

5

0

0

4

4

1

1

0

0

5

5

3

3

18

0

18

0

8

8

3

3

1

0

2

0

0

0

24

4

4

4

18

0

0

0

0

0

0

0

0

0

0

0

0

0

0

0

0

0

0

0

0

0

18

12

0

6

0

0

0

0

0

0

0

0

0

0

0

0

0

0

0

0

0

0

0

0

0

0

0

0

0

0

0

0

0

2

0

0

0

0

2

2

0

0

3629

0

0

0

0

0

0

0

0

0

0

0

928

34

34

38

38

126

126

730

730

152

0

0

0

10

10

26

26

0

0

0

0

0

0

116

0

40

44

6

2

3

0

15

6

0

58

58

58

136

3

3

0

0

133

8

22

103

0

0

0

0

0

0

0

0

768

768

0

0

0

113

450

0

0

6

0

0

110

1

3

0

13

0

72

471

56

56

137

101

36

278

79

60

43

50

0

46

883

1

0

1

31

0

31

489

0

0

489

0

0

77

77

0

0

0

0

149

0

12

12

0

0

0

0

0

0

0

69

39

0

0

0

0

17

136

0

10

41

0

0

5

0

0

0

0

8

25

0

19

28

0

0

0

0

0

0

0

217

217

0

184

0

6

21

6

0

0

0

16

16

16

17

0

0

0

8

6

0

0

6

2

2

0

0

9

0

0

0

0

0

0

9

0

9

0

0

0

155

26

0

0

4

4

2

2

20

3

15

2

16

0

0

16

0

9

7

0

0

0

0

26

2

2

0

0

15

3

12

2

2

7

7

0

4

4

4

28

6

6

7

7

15

0

15

0

55

0

0

48

1

8

8

17

13

1

2

0

1

1

0

5

0

5

0

0

0

0

0

0

0

0

0

0

0

0

0

0

0

0

0

0

0

0

0

0

0

0

0

0

1

1

0

0

0

1

1

1

2

0

0

0

0

0

0

0

0

0

0

2

2

2

2

68

49

0

0

0

0

0

0

0

0

0

0

0

0

0

0

0

0

0

0

0

0

0

0

0

0

0

0

0

49

46

46

46

3

3

3

0

0

0

0

19

19

19

10

8

2

7

7

2

1

1

0

0

0

0

0

0

0

0

0

0

0

0

7925

484

484

2

2

2

298

27

27

0

0

0

271

0

0

271

0

184

0

0

26

0

3

5

18

0

0

25

25

3

3

0

0

0

0

0

0

84

3

81

0

46

1

42

3

0

0

60

60

60

60

60

427

27

26

0

0

0

0

0

0

16

16

0

0

10

10

0

0

1

1

0

1

62

22

22

22

9

9

9

2

2

2

8

8

8

17

17

17

4

4

4

23

0

0

0

0

0

0

2

2

2

0

0

0

19

19

19

2

2

2

0

0

0

0

315

6

0

0

0

6

0

6

0

0

0

0

56

56

0

3

53

190

20

6

14

164

3

161

6

2

4

63

0

0

5

5

58

58

2919

1972

5

5

5

772

618

0

300

3

59

38

183

35

1

1

2

0

2

93

10

1

82

33

12

4

17

25

25

1195

21

21

0

0

1171

1171

0

0

3

3

947

640

640

109

508

23

307

1

1

11

11

295

295

3936

0

0

0

0

3

3

3

3

0

0

0

0

0

0

0

0

0

0

0

0

67

67

1

1

0

0

9

6

3

8

8

1

1

0

0

23

4

3

3

13

17

0

9

8

4

4

0

0

0

0

4

4

10

10

0

0

0

0

6

6

4

4

123

63

63

0

61

0

2

0

0

56

56

15

18

11

12

4

1

1

0

0

0

3

3

0

0

89

89

33

33

0

45

0

12

6

0

0

11

10

6

11

11

164

44

6

6

30

30

8

8

9

1

1

8

8

14

14

8

6

7

7

7

12

12

2

10

78

12

12

21

21

45

45

15

4

4

0

4

0

0

11

0

0

11

11

0

1764

1764

117

106

0

11

1647

18

389

0

0

1

404

804

19

0

0

1

7

4

12

12

12

12

0

0

0

12

12

2

2

10

1

0

8

0

1

0

0

771

675

86

86

2

2

0

0

28

28

1

1

0

0

0

1

0

1

394

0

387

7

163

163

96

14

0

5

9

0

0

67

0

0

8

4

4

0

2

4

0

1

0

0

9

1

3

4

1

0

3

0

13

1

9

9

9

0

0

0

6

6

102

102

0

0

0

0

5

3

1

0

1

8

8

86

2

8

15

9

39

13

0

0

0

0

0

2

2

0

0

1

1

804

385

0

0

173

173

50

3

47

0

0

2

0

2

105

0

46

35

21

3

4

4

2

2

49

49

31

3

3

3

3

1

1

3

0

3

18

1

17

3

3

0

0

0

0

0

82

78

72

0

6

4

4

271

8

8

16

16

10

10

12

0

12

0

0

1

1

6

6

4

4

0

0

0

0

0

0

15

15

189

3

20

166

7

7

2

2

0

0

1

1

1

1

1

34

0

0

34

34

99

99

99

99

1

53

44

1

79

79

79

79

0

0

79

0

0

3

0

0

16

18

0

0

0

19

4

0

0

9

0

0

0

10

0

0

0

0

0

0

0

0

0

0

0

0

0

0

0

0

0

0

195

195

195

195

195

195

1233

1233

1233

857

10

10

0

0

0

0

847

810

37

376

376

250

24

44

9

49

0

0

0

0

717

717

717

139

139

0

46

93

0

0

565

565

42

523

0

0

0

0

0

0

0

13

13

13

12

0

0

0

0

0

0

0

0

0

0

0

0

0

0

0

12

12

5

5

5

2

2

2

5

5

5

12

12

12

12

5

2

3

7

7

457

209

14

14

14

14

195

74

57

52

5

2

2

3

3

12

12

121

3

3

36

36

6

6

76

76

120

83

83

83

32

35

13

3

3

0

0

0

3

3

3

34

34

2

2

14

2

0

12

8

6

2

0

0

0

0

10

5

2

3

8

1

1

1

1

7

7

7

7

0

0

0

0

17

17

17

0

0

17

0

0

5

0

3

0

1

8

0

0

0

0

0

103

103

5

5

5

77

77

77

21

21

21

0

0

0

0

370

3

3

3

3

3

0

0

0

0

0

0

0

0

0

0

0

0

0

0

0

0

0

0

0

0

0

0

0

0

0

0

0

0

0

0

0

0

0

0

0

0

0

0

0

0

0

0

0

0

0

0

0

0

0

0

0

0

0

0

0

0

0

0

0

0

0

0

0

367

367

8

8

8

0

0

0

42

42

42

0

0

0

0

0

24

24

24

291

35

35

16

11

5

0

0

55

55

131

131

54

54

1

1

1

1

1

1

0

0

0

0

0

0

0

0

0

0

0

0

0

0

0

625

625

625

625

71

71

554

147

365

42

0

0

0

0

37

24

11

11

4

4

7

5

2

0

0

0

0

13

12

12

12

1

1

1

0

0

0

0

0

0

0

0

0

0

0

0

11

11

11

11

11

2

1

1

1

1

0

0

0

1

1

1

0

1

14

14

14

9

9

0

9

0

5

0

0

0

5

2

0

0

0

0

1

2

1

1

1

1

0

0

1

1

22516

6408

13

13

2

2

0

5

5

6

0

1

0

1

0

4

19

19

12

12

7

7

159

5

5

5

2

2

2

152

72

72

80

80

14

14

14

3

11

0

3126

3122

0

0

0

0

13

12

1

0

0

45

5

40

0

0

0

58

44

0

14

5

5

1

1

0

29

29

0

0

0

0

5

5

0

0

6

6

0

0

8

0

0

8

0

0

130

0

1

0

1

0

1

0

0

0

64

13

2

20

0

8

0

9

6

3

2

26

26

0

0

4

1

3

0

0

12

0

10

2

892

353

1

32

504

2

0

94

0

0

0

37

55

0

1

1

0

17

17

481

37

0

0

438

6

30

30

0

0

106

106

1

1

0

7

7

2

0

2

29

29

70

70

24

24

125

1

3

36

85

0

0

6

6

34

34

0

0

13

13

21

10

11

0

0

0

11

4

7

24

24

0

771

14

116

2

8

3

41

510

5

0

27

45

0

22

22

0

4

4

4

0

0

0

0

2248

1383

561

561

76

6

16

54

1

0

1

0

0

237

237

0

178

178

316

275

4

0

5

32

14

14

5

5

5

689

14

14

0

0

624

624

0

0

18

18

0

0

33

33

0

171

0

0

12

0

12

0

0

0

0

10

10

149

149

22

22

0

0

0

0

22

5

0

1

2

12

0

2

0

0

41

8

8

8

0

0

0

0

0

0

2

2

2

31

31

31

0

0

0

0

0

1

1

1

1

743

349

22

0

22

0

0

131

131

1

1

0

0

0

0

0

45

0

45

0

0

11

11

0

0

26

19

2

3

2

6

1

0

0

5

0

16

0

0

0

9

0

0

0

7

0

2

2

0

7

7

16

0

16

66

1

1

0

64

69

69

69

6

6

6

15

2

0

2

5

1

0

0

4

8

8

2

2

2

1

1

1

7

3

3

4

4

294

63

5

4

0

1

50

3

22

22

0

0

13

6

7

0

0

0

0

55

14

13

10

18

137

136

1

0

0

4

2

2

0

0

0

22

11

11

11

0

0

0

11

11

11

0

0

0

0

0

0

11909

0

0

0

0

1067

300

9

9

8

8

22

22

33

27

1

5

0

0

139

9

130

17

17

68

68

4

4

0

0

767

0

0

0

0

0

0

0

767

24

0

0

0

4

11

0

0

0

667

23

1

18

0

0

17

2

0

0

0

0

0

0

0

0

0

0

0

0

0

1187

356

0

0

0

0

97

97

193

98

0

0

0

87

8

0

0

2

2

57

35

22

7

7

831

6

6

66

66

131

46

85

26

0

26

243

198

45

3

3

13

13

151

151

120

20

100

10

10

31

31

0

0

23

23

8

8

0

0

0

0

0

0

0

0

326

63

1

1

4

4

58

11

47

263

263

261

2

0

0

0

0

0

0

148

148

0

0

0

0

139

3

5

5

0

0

6

18

0

8

70

0

0

0

0

0

0

0

0

2

2

13

0

0

0

7

0

0

0

3

3

6

4

2

0

12

2

2

2

0

0

0

2

2

2

8

8

8

367

367

367

367

0

8777

6

6

2

4

168

5

5

0

0

162

162

1

1

0

0

7928

7924

0

1188

89

134

5060

0

889

0

257

38

269

0

0

4

4

0

13

13

0

13

606

10

10

3

3

5

3

2

0

1

0

0

1

267

0

51

1

0

0

215

2

2

234

176

0

0

0

0

58

6

6

71

0

2

69

4

2

2

2

2

1

1

0

0

0

0

56

56

56

0

0

0

0

20

20

20

0

0

19

0

1

0

5

5

0

0

0

0

5

5

0

0

0

0

0

0

0

0

0

0

2

2

2

2

2

4197

14

14

0

0

14

14

4183

0

0

0

1878

0

0

0

0

0

0

19

9

10

1859

252

1007

19

84

0

179

0

167

151

2290

2104

2104

0

128

128

58

3

55

0

0

0

0

0

15

15

15

0

0

0

0

0

53

6

3

3

3

3

0

0

0

0

0

2

2

2

2

1

1

1

1

0

0

0

0

0

0

2

2

2

2

2

0

0

0

0

0

0

0

0

0

0

0

18

1

1

1

1

8

8

0

0

0

1

0

1

7

7

0

0

0

0

0

0

0

4

4

4

4

0

0

0

0

5

5

5

5

0

0

0

0

0

0

27

27

27

27

27

38

38

38

38

38

25

13

6

6

6

6

6

5

1

0

0

0

0

0

0

91

91

91

11

11

11

13

13

13

67

67

67

0

0

0

0

0

0

0

0

0

0

0

0

0

0

0

0

1

0

0

0

0

0

0

0

0

0

1

1

1

1

0

0

1

1

0

0

0

0

0

0

0

0

0

0

0

0
